# DNA methyltransferase 1 mutations and mitochondrial pathology: is mtDNA methylated?

**DOI:** 10.3389/fgene.2015.00090

**Published:** 2015-03-12

**Authors:** Alessandra Maresca, Mirko Zaffagnini, Leonardo Caporali, Valerio Carelli, Claudia Zanna

**Affiliations:** ^1^Unit of Neurology, Department of Biomedical and NeuroMotor Sciences, University of BolognaBologna, Italy,; ^2^Department of Pharmacy and Biotechnology, University of BolognaBologna, Italy,; ^3^IRCCS Institute of Neurological Sciences of BolognaBellaria Hospital, Bologna, Italy

**Keywords:** DNMT1 mutations, mtDNA methylation, ADCA-DN, HSN1E, mitochondrial dysfunction

## Abstract

Autosomal dominant cerebellar ataxia-deafness and narcolepsy (ADCA-DN) and Hereditary sensory neuropathy with dementia and hearing loss (HSN1E) are two rare, overlapping neurodegenerative syndromes that have been recently linked to allelic dominant pathogenic mutations in the *DNMT1* gene, coding for DNA (cytosine-5)-methyltransferase 1 (DNMT1). DNMT1 is the enzyme responsible for maintaining the nuclear genome methylation patterns during the DNA replication and repair, thus regulating gene expression. The mutations responsible for ADCA-DN and HSN1E affect the replication foci targeting sequence domain, which regulates DNMT1 binding to chromatin. DNMT1 dysfunction is anticipated to lead to a global alteration of the DNA methylation pattern with predictable downstream consequences on gene expression. Interestingly, ADCA-DN and HSN1E phenotypes share some clinical features typical of mitochondrial diseases, such as optic atrophy, peripheral neuropathy, and deafness, and some biochemical evidence of mitochondrial dysfunction. The recent discovery of a mitochondrial isoform of DNMT1 and its proposed role in methylating mitochondrial DNA (mtDNA) suggests that *DNMT1* mutations may directly affect mtDNA and mitochondrial physiology. On the basis of this latter finding the link between DNMT1 abnormal activity and mitochondrial dysfunction in ADCA-DN and HSN1E appears intuitive, however, mtDNA methylation remains highly debated. In the last years several groups demonstrated the presence of 5-methylcytosine in mtDNA by different approaches, but, on the other end, the opposite evidence that mtDNA is not methylated has also been published. Since over 1500 mitochondrial proteins are encoded by the nuclear genome, the altered methylation of these genes may well have a critical role in leading to the mitochondrial impairment observed in ADCA-DN and HSN1E. Thus, many open questions still remain unanswered, such as why mtDNA should be methylated, and how this process is regulated and executed?

## DNA Methylation

Several epigenetic signals participate in cell specific gene expression, including DNA methylation and demethylation, post-translational modifications of histone proteins (i.e., acetylation, methylation, phosphorylation, and ubiquitination), incorporation of histone variants and gene regulation by non-coding RNAs (Rivera and Ren, 2013; Kanherkar et al., 2014a). DNA methylation, which occurs in all prokaryotic and eukaryotic organisms, with rare exception for yeast, roundworm, and fruit fly, is a key epigenetic process involved in the regulation of gene expression (Lande-Diner et al., 2007; Lee et al., 2010) and parental imprinting (Sha, 2008; Ishida and Moore, 2013), in chromosome X inactivation (Straub and Becker, 2007) as well as in the development of the immune system (Cedar and Bergman, 2011; Kondilis-Mangum and Wade, 2013; Li et al., 2013) and in cellular reprogramming (Reik, 2007; Krishnakumar and Blelloch, 2013; Papp and Plath, 2013; Kanherkar et al., 2014b). Furthermore, it is engaged in the maintenance of the genome integrity through protection against endogenous retroviruses and transposons (Howard et al., 2008). In humans aberrant DNA methylation patterns are associated with several diseases, including various cancers (Jones and Baylin, 2007; Esteller, 2008; Iacobuzio-Donahue, 2009), immune system disorders (Feinberg, 2007) and neurodegeneration (Jakovcevski and Akbarian, 2012; Qureshi and Mehler, 2013).

### Classical Model of DNA Methylation

In prokaryotes, DNA methylation occurs on both cytosine and adenine bases and is one of the host restriction systems to distinguish self and non-self DNA (Jeltsch, 2006). In mammals, following the classical model of DNA methylation, it takes place in the cytosine residues at their C5 positions, primarily in the CG dinucleotides (CpG), acting mainly as a repressive tag to silence chromatin and inhibit transcription. In the human genome there are 56 million CG sites, about 60–80% of which are methylated, corresponding to 4–6% of all cytosines (Laurent et al., 2010). Methylation levels and patterns are cell and tissue specific. In mammalian genomes, the CpG are poorly represented compared to other dinucleotides, because of the higher mutagenic property of the 5-methyl-cytosine base compared to the unmethylated one (Pfeifer et al., 2000). The irregular CpG distribution is reflected by their depletion in intergenic and intragenic sequences, whereas their presence is less suppressed in repetitive DNA (such as transposons and retroviruses) and CpG islands, regions of at least 550 bp, with a ratio of observed CpG/expected CpG higher than 0.65 (Takai and Jones, 2004). Around 70% of human genes promoter regions present the CpG islands and an inverse correlation between CpG islands density and the promoter methylation status exists. Moreover, active genes usually show hypomethylation at the transcriptional start site (TSS) and high levels of methylation in the gene body, which is supposed to block aberrant transcription initiation inside the gene, avoiding the production of truncated mRNAs and proteins. The splicing sites are regions characterized by a change in DNA methylation, since exons have higher methylation than introns, suggesting a strong implication of DNA methylation in the splicing process (Laurent et al., 2010; Chatterjee and Vinson, 2012).

### DNA Methyltransferase Enzymes

**FIGURE 1:**
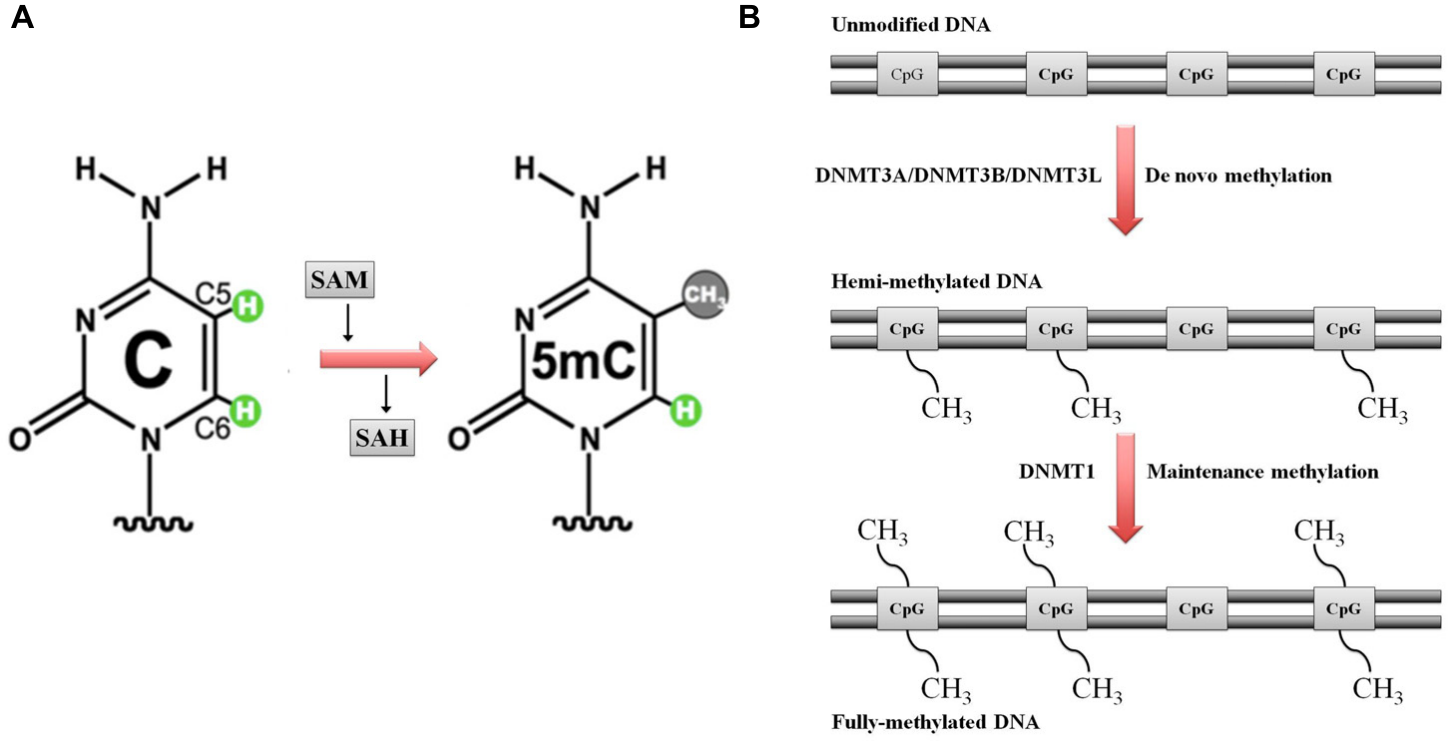
Schematic representation of DNA methylation. **(A)** DNA (cytosine-5)-methyltransferases (DNMTs) transfer the methyl group at the DNA cytosine ring carbon C5 by using *S*-adenosyl methionine (SAM) as methyl donor. *S*-adenosyl homocysteine (SAH) is the cofactor product. **(B)** DNA methylation: classical model. Maintenance versus *de novo* methylation. Figure modified by Cheng and Blumenthal ().

**FIGURE 2:**
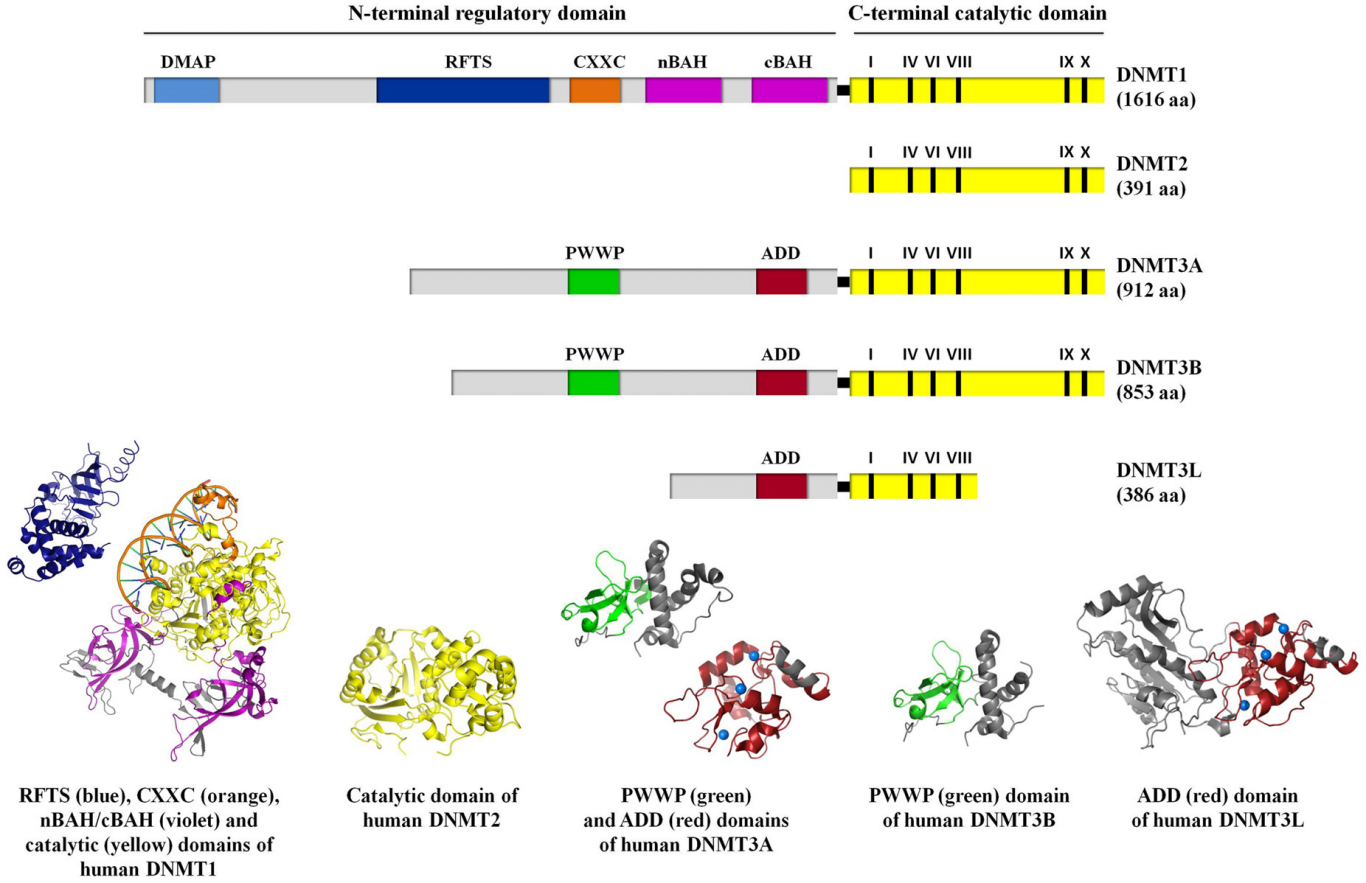
A schematic representation of the domain structure of human DNMT isoforms. For human DNMT1, DNA methyltransferase associated protein (DMAP), replication foci targeting sequence (RFTS), CXXC, bromo-adjacent homology (nBAH/cBAH), and catalytic domains are represented by cyan, blue, orange, violet, and yellow bars, respectively. For human DNMT2, catalytic domain is represented by yellow bar. For human DNMT3A, DNMT3B, and DNMT3L, PWWP, ADD, and catalytic domains are represented by green, red, and yellow bars, respectively. In the catalytic domain (yellow bar), the conserved C5 DNA MTase motifs in the C-terminal part of each DNMT isoforms are labeled. The interdomain linker regions are shown as light gray bars. Numbers in parentheses indicate the length of each protein. The crystal structures of the human DNMT1 RFTS domain (residues 351–597, PDB ID: 3EPZ; Syeda et al., 2011), human DNMT1 CXXC, nBAH/cBAH, and catalytic domains bound to DNA-containing unmethylated CpG sites (residues 646–1600, PDB ID: 3PTA; Song et al., 2011), human DNMT2 catalytic domain (residues 1–188 and 248–391, PDB ID: 1G55; Dong et al., 2001), human DNMT3A PWWP (residues 275–427, PDB ID: 3LLR), and ADD domains (residues 476–614, PDB ID: 3A1A; Otani et al., 2009; Wu et al., 2011, respectively), human DNMT3B PWWP domain (residues 293–442; PDB ID: 3QKJ; Wu et al., 2011), human DNMT3L ADD domain (residues 34–277 and 279–380, PDB ID: 2PV0; Ooi et al., 2007), are shown. For further details see “Text."

DNA methylation is catalyzed by a group of enzymes called DNA (cytosine-5)-methyltransferases (DNMTs) that transfer a methyl group from a cofactor molecule *S*-adenosyl-l-methionine (AdoMet or SAM) to the C5 position of the cytosine residues to generate 5-methylcytosine (5 mC) and *S*-adenosyl-l-homocysteine (AdoHcy, SAH; **Figure 1**). The mammalian DNMT family includes four members: DNMT1, DNMT3A, DNMT3B, and DNMT3L. These enzymes comprise two parts: a C-terminal catalytic portion and a large N-terminal region of variable size containing regulatory domains involved in the interaction with DNA, chromatin, and other proteins (**Figure 2**). Furthermore, the N-terminal region contains 621 amino acids required for discriminating between hemi-methylated and unmethylated DNA. The C-terminal catalytic domain, being highly conserved between eukaryotes and prokaryotes, is composed by 500 amino acids and harbors the active center of the enzyme, which contains amino acids motifs characteristic of the cytosine-C5 methyltransferases, called the “AdoMet-dependent MTase fold" (Jurkowska et al., 2011a). Motifs I and X of this domain are involved in cofactor binding whereas motifs IV, VI, and VIII have a catalytic function. The non-conserved region between motifs VIII and IX is the target recognition domain (TRD), crucial for DNA recognition and specificity (Cheng, 1995; Jeltsch, 2002; Cheng and Blumenthal, 2008). Considering the high variability of the N-terminus of DNTMs, a detailed description of this region for each DNMT isoforms will be provided in the following paragraphs.

The initial methylation pattern is established by *de novo* DNMTs (DNMT3 family in mammals). This pattern is perpetuated for the rest of the life (with small tissue-specific changes) by a mechanism first proposed by Holliday and Pugh (1975) and Riggs (1975).

Each round of DNA replication produces hemimethylated DNA with the methylation marks in the parental stand and the new synthesized daughter unmethylated strand (**Figure 1B**). The maintenance DNA methyltransferase (DNMT1 in mammals), showing a preference for hemimethylated sites, copies the existing methylation pattern (Jones and Liang, 2009; Denis et al., 2011).

### DNMT3 Family

During the embryogenesis the DNMT3 family establishes the initial CpG methylation pattern (Chen and Li, 2006; Jurkowska et al., 2011a). The DNMT3 family includes two active *de novo* DNMTs, DNMT3A, and DNMT3B, without significant preference between hemimethylated and unmethylated DNA, and one catalytically inactive regulatory factor, the DNMT3-Like protein (DNMT3L; Bestor, 2000; Jurkowska et al., 2011a).

Knockout of DNMT3A or DNMT3B is lethal, being both essential for embryonic development in mice. Mouse DNMT3B knockout embryos die *in utero*, whereas the DNMT3A knockout animals die shortly after birth (Okano et al., 1999). DNMT3L knockout mice are viable, but male are sterile, failing to produce mature sperm (Hata et al., 2002). Mutations in the human *DNMT3B* gene are associated with a rare autosomal disease, called ICF (Immunodeficiency, Centromere instability, Facial abnormalities) syndrome, which is accompanied by hypomethylation of classical satellites of the pericentromeric regions of chromosomes 1, 9, and 16, probably due to reduction of enzyme catalytic efficiency or alteration of its localization (Xu et al., 1999; Weemaes et al., 2013). *De novo* mutations in *DNMT3A* have also been identified in patients with overgrowth disorders (Tatton-Brown et al., 2014). Despite high sequence homology and similar biochemical properties, DNMT3A and 3B show partially non-overlapping biological functions with DNMT3A being involved in the setting of parental imprints and DNMT3B in the methylation of pericentromeric repeats (Okano et al., 1999; Kaneda et al., 2004).

From the structural point of view, the DNMT3 enzymes contain a N-terminal variable portion (around 280 and 220 amino acids for DNMT3A and DNMT3B, respectively), followed by two conserved regions: the regulatory PWWP and ADD domains and C-terminal catalytic portion. DNMT3L lacks the PWWP domain as well as the DNMTs motifs IX and X and all the important catalytic residues in its C-terminal portion (**Figure 2**). The PWWP is a variable module of 100–150 amino acids characterized by the presence of a strictly conserved proline-tryptophan-tryptophan-proline motif, also found in chromatin associated eukaryotic protein from yeast to mammals. It comprises a globular domain with a five-stranded β-barrel followed by a five-helix bundle (**Figure 2**; Qiu et al., 2002; Chen et al., 2004). *In vitro* experiments showed that the PWWP domain of DNMT3B mediate DNA binding, but it is not required for CpG methylating activity (Qiu et al., 2002). Subsequently, it has been demonstrated that it exhibits a dual function: binding to both DNA and methylated lysine on histone proteins (Chen et al., 2004; Dhayalan et al., 2010; Wu et al., 2011). The ADD domain (ATRX-DNMT3-DNMT3L), also known as PHD domain (plant homodomain), is present in all the proteins belonging to DNMT3 family and in the ATRX (alpha thalassemia/mental retardation syndrome X-linked) protein. This domain is a cysteine-rich module that binds zinc ions (comprising six CXXC motifs; **Figure 2**) and constitutes a platform for various protein–protein interactions. It has been reported that DNMT3L, which only owns this characteristic motif, recognizes specifically the unmethylated lysine 4 of histone H3 through its ADD domain and induces *de novo* DNA methylation by recruitment or activation of DNMT3A, thanks to an interaction between its C-terminal domain and the catalytic portion of DNMT3A. These data support the idea that DNMT3L has the dual function of binding the unmethylated histone tail and activating DNA methyltransferase (Jia et al., 2007; Ooi et al., 2007). Nevertheless, the ADD domains of DNMT3A and 3B share considerable homology with DNMT3L and it has been reported a direct binding of the DNMT3A and DNMT3B ADD domains to H3 tails unmodified at lysine 4 (Otani et al., 2009; Zhang et al., 2010). Since the ADD domains of DNMT3A/B and DNMT3L clearly show the same binding specificity, the recruitment of DNMT3A to chromatin with unmethylated H3 tail is probably not the primary function of DNMT3L (Zhang et al., 2010).

Jurkowska et al. (2011b) reported that DNMT3A forms oligomers predicted to bind several DNA molecules oriented in parallel and required to tightly tie to heterochromatin by the ADD and PWWP domains, which recognize H3 tails unmodified at lysine 4 and H3 trimethylated at lysine 36, respectively (Dhayalan et al., 2010; Zhang et al., 2010). In addition, the association of the DNMT3A oligomers to DNMT3L changes its subnuclear localization, from heterochromatin to euchromatin, thereby increasing its availability and DNA methylation activity for the generation of DNA methylation imprints (Jurkowska et al., 2011b).

### DNMT1

Whereas DNMT3 family works as *de novo* methylases, DNMT1 maintains the existing pattern of methylation during chromosomes replication (Chen and Li, 2006; Jurkowska et al., 2011a) and repair (Mortusewicz et al., 2005; Loughery et al., 2011). Human DNMT1, a large enzyme of 1616 amino acids, is the most abundant DNMT involved in preserving and propagating the existing methylation patterns during cell division (Jurkowska et al., 2011a; Kar et al., 2012). It shows a preference for hemimethylated DNA (Goyal et al., 2006) and it is localized at the DNA replication foci during the S-phase of cell cycle (Leonhardt et al., 1992). In addition, DNMT1, being a highly processive enzyme, is able to methylate long stretches of DNA without dissociation (Hermann et al., 2004; Vilkaitis et al., 2005). These two features make it suitable for its role in the maintenance of methylation patterns. The expression of DNMT1 is ubiquitous and high in proliferating cells and varies in a cell-cycle-dependent manner, being maximal during the S-phase and extremely low in non-dividing cells (Robertson et al., 1999; Kimura et al., 2003). Concerning the subcellular localization of DNMT1, it is diffusely distributed in the nucleus during the interphase and moves to the replication foci for early and mid S-phase creating a characteristic punctuate pattern (O’Keefe et al., 1992; Easwaran et al., 2008). *DNMT1* mouse knockout shows extensive demethylation of the genome and embryonic lethality shortly after gastrulation, underlying the crucial role of DNMT1 in early development. Embryonic stem cells lacking DNMT1 are viable, despite the low level of DNA methylation, but die after differentiation induction (Li et al., 1992; Chen et al., 1998). Complete inactivation of DNMT1 in human colorectal carcinoma cells leads to severe mitotic defects and progressive cell death (Chen et al., 2007).

Mutations affecting the *DNMT1* gene have been associated with two distinct autosomal dominant neurodegenerative diseases: hereditary sensory and autonomic neuropathy with dementia and hearing loss type 1E (HSN1E) and autosomal dominant cerebellar ataxia-deafness and narcolepsy (ADCA-DN; Klein et al., 2011; Winkelmann et al., 2012). All these mutations are localized in the DNA replication foci targeting sequence (RFTS) domain, essential in mediating the association of DNMT1 to heterochromatin. Mutations in exon 20 of *DNMT1* have been associated with HSN1E, whereas mutations in exon 21 have been found in ADCA-DN (Klein et al., 2013).

From the structural point of view, DNMT1 is a multi-domain enzyme composed by a N-terminal regulatory region and a C-terminal catalytic domain joined by a series of KG (lysine-glycine) repeats (**Figure 2**; Dhe-Paganon et al., 2011; Jurkowska et al., 2011a; Kar et al., 2012). The N-terminal portion contains several motifs:

• DMAP1 (DNA methyltransferase associated protein 1) charged rich domain is involved in the interaction of DNMT1 with the transcriptional repressor DMAP1 as well as in the stability of the enzyme and in its binding to DNA CpG sites (Rountree et al., 2000; Ding and Chaillet, 2002).
• PBD (PCNA-proliferating cell nuclear antigen-binding) domain address DNMT1 to the replication foci through the interaction between DNMT1 and PCNA.).
• NLS (nuclear localization sequence) are at least three.
• RFTS (replication foci targeting sequence) is crucial for the localization of DNMT1 at the centromeric chromatin and replication foci as well as for its dimerization (Easwaran et al., 2008; Fellinger et al., 2009).
• CXXC zinc domain, similar to the cysteine-rich motif present in other chromatin associated proteins. It contains eight conserved cysteine residues clustered in two CXXCXXC repeats that bind to two zinc ions and is necessary for the recognition of unmethylated CpG, thus influencing the catalytic activity of the enzyme (Pradhan et al., 2008).
• PBHD (polybromo homology domain) domain is composed by the BAH1 and BAH2 (Bromo-adjacent homology 1 and 2) motifs, typical of proteins involved in transcriptional regulation. It has been proposed to act as a protein–protein interaction module specialized in gene silencing even if its functional role in DNMT1 remain unknown.

Thus, the N-terminal regulatory region is designed to recognize the methylation target site (DMAP1, PBD, and RFTS domains), to bind nucleic acids trough an allosteric site (CXXC domain) and to allow interaction with other proteins (PBHD domains), making DNMT1 ideally suited for its role as the maintenance methyltransferase.

The KG linker between the N- and C-terminal regions of the enzyme is composed by a series of lysine and glycine residues, which might contribute to address DNMT1 to the region next to the replication fork.

Finally, the C-terminal region encloses the catalytic center of the enzyme bearing the conserved motifs I–X, and is folded in large and small domains, separated by a huge cleft. The large domain (motifs I-VIII and part of motif X) participates in SAM cofactor binding substrate (cytosine) targeting and other essential catalytic events. The small domain includes a variable region between motif VIII and IX, named TRD (target recognition domain), the conserved motif IX and part of motif X. The catalytic domain allows the binding of the target DNA in the active site and various other regulatory molecules in the allosteric sites, which support a multiple levels of enzyme regulation (Dhe-Paganon et al., 2011; Jurkowska et al., 2011a; Kar et al., 2012).

In order to avoid runaway methylation, DNMT1 undergoes to several auto-inhibitory mechanisms. In particular, to keep DNMT1 inactive the RFTS domain is positioned and stabilized deep inside the catalytic domain in a task where the hemimethylated DNA is expected to fit, thus, masking the catalytic core of the enzyme (Qin et al., 2011; Syeda et al., 2011; Bashtrykov et al., 2014). The auto-inhibitory function of the CXXC domain is more controversial. Song et al. (2011). reported that CXXC specifically binds to unmethylated CpG, activating the CXXC-BH21 linker, which then occupies the catalytic pocket and interferes with its function. By contrast, Bashtrykov et al. (2012) refused this auto-inhibitory mechanism, proposing instead that the recognition of the hemimethylated state of target sites resides within the catalytic domain. While the unmethylated DNA emerges from the replication fork, DNMT1 is tethered and kept auto-inhibited to avoid unauthorized *de novo* methylation of the DNA. As soon as the hemimethylated sites are released from the replication complex, the TRD region of DNMT1 in combination with UHRF1 (ubiquitin-like with PHD and ring finger domains 1) recognizes and marks them as prime targets for methylation to guarantee the correct methylation patterns across successive generations (Kar et al., 2012).

### DNMT2

DNA (cytosine-5)-methyltransferases 2 is the smallest mammalian DNMT, now named TRDMT1, which has the potential to methylate RNA instead of DNA (Goll et al., 2006; Schaefer and Lyko, 2010). It completely lacks the regulatory N-terminal region and the C-terminal domain contains all 10 sequence motifs that are conserved among DNMTs, including the consensus *S*-adenosyl-l-methionine-binding motifs (Okano et al., 1998; Dong et al., 2001; **Figure 2**). Despite the high sequence and structural similarity with other DNMTs and its capability to bind the DNA, DNMT2 has failed to show a significant transmethylase activity (Hermann et al., 2003). Nonetheless, it methylates cytosine 38 in the anticodon loop of tRNA^Asp^ (Goll et al., 2006), by using a DNA methyl transferase-like catalytic mechanism (Jurkowski et al., 2008). Thus, DNMT2 seems to have intermediate properties between a DNA methyl transferase and a RNA methyl transferase, with which it shares the structural/catalytic features and the nuclear-cytoplasmic localization, respectively (Schaefer et al., 2008).

### Stochastic Model of DNA Methylation

For long time the classical model of DNA methylation at CpG sites in mammals, with the involvement of both DNMT3 and DNMT1 (i.e., *de novo* and maintenance DNA methyltransferases, respectively), has been considered a paradigm for epigenetic information transfer (Holliday and Pugh, 1975; Riggs, 1975). Over the years, several experimental observations highlighted that the site-specific maintenance methylation model had to be revised. Thus, Jeltsch and Jurkowska (2014) proposed a modified stochastic DNA methylation model which includes a new description of both *de novo* and maintenance methylation process at CpG and non-CpG sites.

An efficient methylation of both DNA strands, starting from unmethylated DNA, can be achieved by the tight cooperation of DNMT3 enzymes with DNMT1. Thus, *de novo* and maintenance methylation could not be regarded as two distinct events. DNMT3 enzymes, and in particular DNMT3A, can bind adjacent DNA molecules, but only one of the two strands is preferentially methylated. By contrast, the hemi-methylated DNA is the favorite substrate for DNMT1, which copies the methylation on the second strand (Fatemi et al., 2002). In addition, DNMT1 shows considerable *de novo* methylation activity on unmethylated DNA both *in vitro* (Goyal et al., 2006) and *in vivo*, as observed in DNMT3A/3B double knockout embryos (Okano et al., 1999). Arand et al. (2012). have confirmed these results by reporting DNMT1-dependent *de novo* methylation in cells with single or combined DNMTs knockout. On the other side, deletion of DNMT3A/3B or DNMT3B alone led to a reduction of maintenance DNA methylation at repetitive elements, despite the presence of functional DNMT1 (Chen et al., 2003; Dodge et al., 2005; Arand et al., 2012).

Thus, neither *de novo* methylation nor maintenance methylation can be exclusively assigned to DNMT3A/3B or DNMT1, respectively, but a synchronized cooperation of all the DNMTs seems crucial.

The classical model of maintenance methylation assumes that the methylation patterns of single CpG sites are stably inherited and that exists for this purpose a perfect enzyme, characterized by a working efficiency of 100%.

In reality, average methylation densities of DNA regions are maintained, instead of exact CpG site-specific methylation patterns (Zhang et al., 2010), and changes in methylation levels occur through stochastic processes (Landan et al., 2012). In fact, despite the 10–40-fold preference of DNMT1 for hemi-methylated DNA, this feature is not enough to copy accurately the site-specific methylation status of around 56 million CpG sites in the human genome over several rounds of DNA replication (Jeltsch and Jurkowska, 2014).

In addition, patterns of non-CpG DNA methylation, typical of plants, have been reported also in the human genome, taking place in CpA sites, and specifically introduced by DNMT3A. This clearly indicates a permanent *de novo* methylation activity of this enzyme, which is not in agreement with the classical methylation model (Arand et al., 2012; Guo et al., 2014).

Finally, also the DNA demethylation has to be considered in the establishment and maintenance of DNA methylation pattern. This process could be passive, during the replication, or active, performed by the family of ten eleven translocation (TET) dioxygenases. These enzymes oxidize the methyl groups of methylcytosine and are expressed both during early development and also in later stages, suggesting a permanent DNA demethylation. Hydroxymethylation of DNA by TET enzymes has been proposed to keep CpG islands in unmethylated state by counteracting stochastic DNA methylation (Kohli and Zhang, 2013; Wu and Zhang, 2014).

In conclusion, the DNA methylation is influenced by all the events above described, that are combined in the stochastic model of Jeltsch and Jurkowska (2014):

• DNA methylation at each site is determined by the local rates of methylation and demethylation
• The local rates of methylation depend on targeting/regulation of DNMTs/demethylases and on chromatine remodeling/DNA accessibility
• DNA methylation occurs on both CpG and non-CpG sites *de novo* and maintenance methylation are combined in a unified mechanism
• The average DNA methylation level of DNA regions is inherited rather than the methylation state of individual CpG sites.

## Mitochondrial DNA Methylation

Unlike nuclear DNA, the methylation of mtDNA is a highly controversial topic that after 40 years is still matter of debate among researchers. In fact, although recent studies have demonstrated the presence of 5 mC in mtDNA, skepticism regarding the sensitivity and reproducibility of the methods used and the putative biological function of methylation in mitochondria still remains. Indeed, the observed methylation levels are very low (1–5%) and, furthermore, mtDNA has a different organization compared to nuclear DNA (i.e., no histones, lack of introns, multicopy genomes), which should imply a different regulatory mechanism of gene expression through DNA methylation. In fact, mammalian cells typically contain 1,000–10,000 copies of mtDNA, which are organized into nucleoprotein complexes termed nucleoids, being TFAM (mitochondrial transcriptor factor A) the main protein component. The TFAM/mtDNA ratio finely regulates the fraction of active mtDNA molecules available for mitochondrial replication/transcription (Bogenhagen, 2012; Campbell et al., 2012; Farge et al., 2014).

The first approaches to investigate mtDNA methylation date back to 1970s, when Vanyushin et al. (1971) found a DNMT activity in mitochondria isolated from loach embryos, demonstrated the presence of 5 mC in mtDNA extracted from beef heart (Vanyushin and Kirnos, 1974) and evidenced a different specificity for DNA methylases isolated from nucleus and mitochondria, being the mitochondrial specific for mono-pyrimidines and the nuclear for di- and tri-pyrimidines (Vanyushin and Kirnos, 1977). However, in the same years the absence of 5 mC in mtDNA from mouse, hamster, frog, and HeLa cells was also reported (Nass, 1973; Dawid, 1974). Shmookler and Goldstein (1983) and Pollack et al. (1984), studying both human fibroblasts and mouse fibroblastoid cells, clarified that methylation occurred in mtDNA with a frequency of 1.5–5% and only in CpG di-nucleotides, which, however, are underrepresented in mtDNA (Pollack et al., 1984; Cardon et al., 1994). After many years, in 2004, the presence of methylated cytosines in mtDNA, from gastric and colorectal cancers, was once again denied using bisulfite-PCR-single-stranded DNA conformation polymorphism on three selected regions of mtDNA containing 37 CpG sites (Maekawa et al., 2004).

Rebelo et al. (2009) failed to observe mtDNA methylation in human osteosarcoma 143B, HEK293, and HeLa cells, under standard culture conditions, but the same authors also reasoned that percentages of methylation <5% have not been detected by the approach employed (site-specific methylation restriction enzymes). However, in this study, some methylation of mtDNA was observed after the induced-expression of two different mitochondria-targeted bacterial methyltransferases and, additionally, the levels of this methylation increased during mtDNA replication, when it is assumed that nucleoids are remodeled and mtDNA could be less protected by proteins (i.e., TFAM) and more accessible to DNMTs (Rebelo et al., 2009).

The issue of mtDNA methylation came back again in 2011, when two different groups demonstrated the presence of 5 mC and DNMTs in mitochondria through novel and more sensitive approaches (Chestnut et al., 2011; Shock et al., 2011). Surprisingly, a mitochondria targeted DNMT1 isoform (mtDNMT1), conserved in different species, has been found by Shock et al. (2011); the mtDNMT1 seems to be translated from an unconventional ATG site, located immediately upstream of the canonical one, and localized to mitochondria where it bounds to mtDNA. Moreover, the mtDNMT1 gene expression was induced by NRF1 and PGC1α, two master regulators of mitochondrial biogenesis, in HCT116 cells (Shock et al., 2011). Interestingly, gene expression of mtDNA-encoded genes was altered after mtDNMT1 over-expression, with reduced levels of *MT-ND6* in the L-strand and increased levels of *MT-ND1* in the H-strand, suggesting an opposite role for mtDNMT1, and cytosine methylation in the two strands; however, the expression levels of *MT-CO1* and *MT-ATP6*, both in the H-strand, were unaltered (Shock et al., 2011). The authors speculate that mtDNMT1 interferes with MTERF-dependent transcription termination, inducing an increased transcription of *MT-ND1* through the HSP1, with no effects on the polycistronic mRNA produced by HSP2. Lastly, in this study the presence of 5 mC in mtDNA was demonstrated by a methylated DNA-immunoprecipitation (MeDIP) approach, followed by real time-PCR (Shock et al., 2011).

Concomitantly, Chestnut et al. (2011) demonstrated the presence of DNMT3A in mitochondria isolated from mouse brain and from human motor cortex, whereas DNMT1 was faintly detectable in mitochondria from these tissues. Furthermore, co-localization of 5 mC and mitochondria was shown by immunofluorescence experiments using antibodies recognizing 5 mC and a specific mitochondrial marker (superoxide dismutase isoform 2, SOD2; Chestnut et al., 2011).

The mtDNA methylation was also investigated by a liquid chromatography-electrospray ionization tandem-mass spectrometry (LC-ESI-MS) method, proving the existence of 5 mC in the mitochondrial genome (Infantino et al., 2011), and soon after also 5 hmC have been identified in mtDNA by an ELISA approach (Dzitoyeva et al., 2012).

In a study focused on the intragenic methylation of *PolgA* in different mouse cells/tissues as possibly correlated with PolgA expression and mtDNA content regulation, the authors found in the eluted fraction of MeDIP analysis some mtDNA encoded genes (*mt-cytb*, *mt-co1*) and the D-loop region, indicating that these genes have 5 mC and 5 hmC, although at very low levels (Kelly et al., 2012).

Pirola et al. (2013) further evidence of methylated mtDNA has been published. The methylation of *MT-ND6*, *MT-CO1* and of the D-loop of mtDNA was assessed by quantitative methylation specific-PCR in the context of non-alcoholic fatty liver disease. The authors found a significant association between the condition of non-alcoholic steatohepatitis (NASH) and the methylation of *MT-ND6* gene, which inversely correlates with *MT-ND6* transcription and protein expression in the liver of subject affected by NASH (Pirola et al., 2013).

The methylation of the D-loop region was also confirmed in mammals by bisulfite-sequencing (30 clones for each sample sequenced) and by MeDIP (Bellizzi et al., 2013). Methylation was limited to the L-strand of the D-loop and the majority of methylated cytosines were located outside the CpG nucleotides; different tissues were analyzed (blood cells, fibroblasts, HeLa cells) and tissue-specific patterns of methylation were identified, HeLa cells showing the highest percentage of methylated cytosines (Bellizzi et al., 2013). In the same study, DNMT1 and DNMT3B expression in mitochondria from HeLa and mouse 3T3-L1 cells was evidenced (Bellizzi et al., 2013).

On the contrary, Wong et al. (2013) proved that in mouse skeletal muscle DNMT1 was not present within the organelle, but probably bound to the outer membrane (detectable in the crude mitochondrial fraction), whereas DNMT3A was present in the pure mitochondrial fraction isolated from both mouse skeletal muscle and spinal cord; DNMT3B was not detectable in any of the mitochondrial preparations. DNMT3A was also present in mitochondria from human cerebral cortex, but not in mitochondria from HEK293 cells, showing a preferential expression in mitochondria from excitable tissues in humans and mouse (Wong et al., 2013). In addition, methylated cytosines in the mouse D-loop and *mt-rnr2* (coding for 16S RNA) were identified by bisulfite treatment and pyrosequencing, and different levels of methylation in brain (highest percentage of 5 mC), liver and testes were shown (Wong et al., 2013). Moreover, as previously observed by Chestnut, the co-localization of 5 mC, and mitochondria by immunofluorescence was confirmed and interestingly, it was observed a co-localization of 5 mC with autophagosome (LC3A positive staining), indicating that mtDNA methylation may be involved in mitophagy (Wong et al., 2013).

Also Byun et al. (2013) carried out bisulfite-pyrosequencing to investigate methylation of three mtDNA regions, *MT-TF* (tRNA phenylalanine gene, two CpG sites), *MT-RNR1* (12S RNA gene, two CpG sites), and the D-loop (three CpG sites), in association with effect of airborne pollutants. The authors were able to detect around 5–6% of 5 mC in the selected regions.

Hong et al. (2013) critically revised all methods employed to quantify methylated mtDNA in the last years. Contextually, these authors established the absence of CpG methylation in human mtDNA performing bisulfite-sequencing and analyzing data of bisulfite-next generation sequencing (NGS) previously published by Akalin et al. (2012) and Hong et al. (2013). Bisulfite-sequencing failed to reveal 5 mC in four selected mtDNA regions (*MT-RNR1*, *MT-RNR2*, *MT-CO2*, *MT-ATP6*) of HEK293 cells, and identified very low frequencies of 5 mC in the same regions of HCT116 and blood cells mtDNA (<0.5%), considered not relevant because comparable to the non-convertion rate of bisulfite; cross-contamination of nuclear DNA sequences of mitochondrial origins (NUMTs; Lascaro et al., 2008) has been also excluded based on the specificity of the analyzed regions for mtDNA sequence (Hong et al., 2013). Comparable levels of 5 mC (<0.5%), both in CpG or not-CpG sites, came from the re-analysis of bisulfite-NGS data on HCT116 cells previously published (Akalin et al., 2012), but the mean coverage obtained of 94x may be not sufficient, considering that HCT116 cells have a mtDNA content of about 4000 copies, as mentioned by the authors (Hong et al., 2013).

Lastly, a comparative analysis of mitochondrial methylomes in 39 different cell lines was carried out extracting public data from the NIH Roadmap Epigenomics project (Ghosh et al., 2014). The re-analyzed data, deriving from MeDIP-sequencing included human brain, breast, blood, penis, and two cell lines, H1 and neurosphere cultured cells; moreover, some tissues were analyzed at different developmental time points. The authors identified tissue- and development-specific pattern of 5 mC methylation in mtDNA, with *MT-ND6* and *MT-ATP6* showing progressive reduction in methylation correlated with brain development. However, also in this case, methylated cytosines resulted underrepresented (<0.5%) and an extremely low minimum coverage of 5x was considered (Ghosh et al., 2014).

**FIGURE 3:**
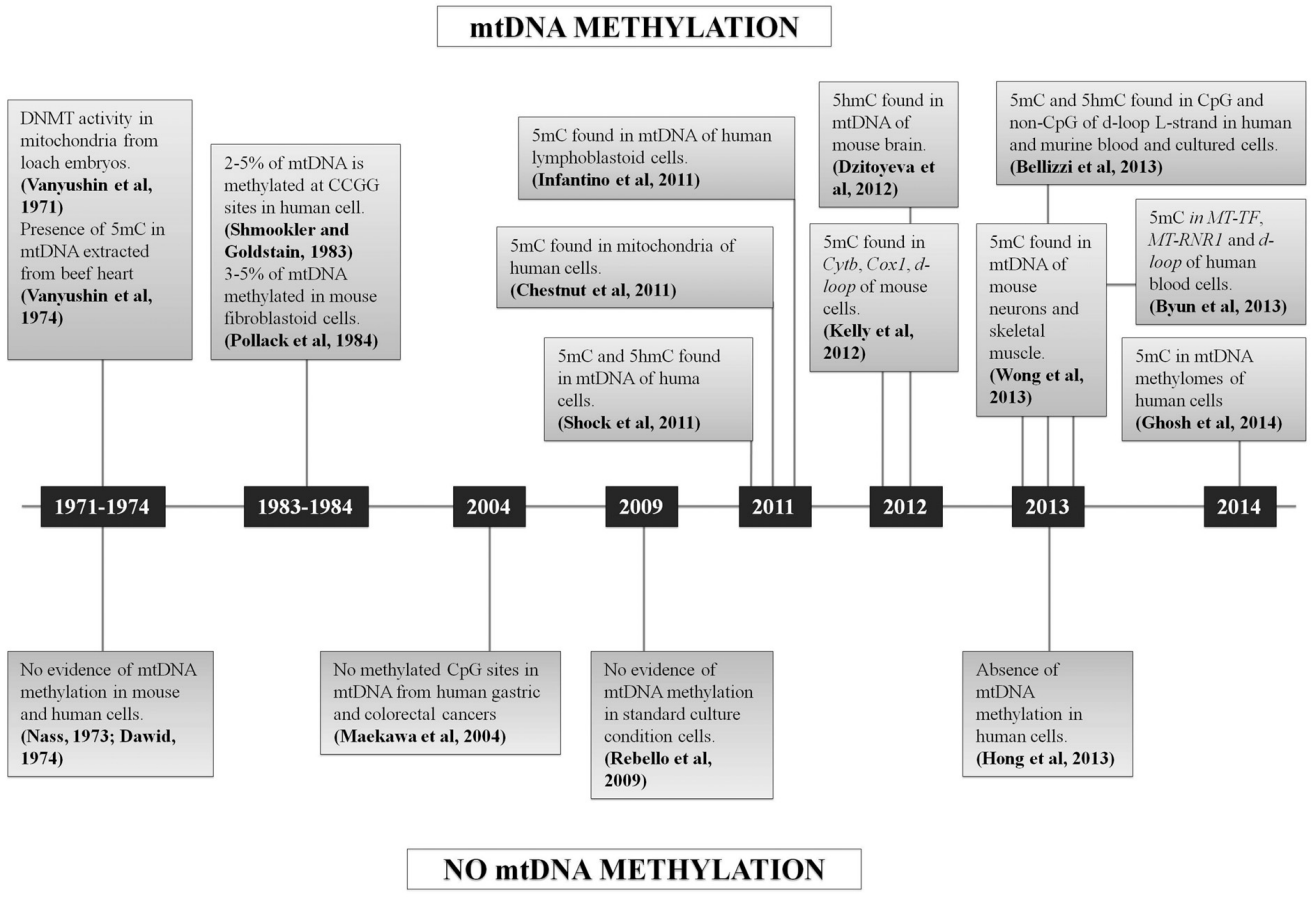
Chronological sequence of mtDNA methylation studies. Schematic representation of all the studies which support or denied the mtDNA methylation.

In conclusion, since 2011 several studies claimed the occurrence of mtDNA methylation, but a few others also asserted the absence of 5 mC in mtDNA (**Figure 3**). Many of the published approaches had some limitations, such as the reproducibility of the methods based on the use of antibodies (ELISA, MeDIP), or the enrichment by amplification before sequencing that may create a bias, or the very low coverage considered for the analysis of NGS data that may influence the quantification of 5 mC referred to total mtDNA copies. In fact, the analysis of mtDNA methylation should take into account that the mitochondrial genome is multicopy, differing for copy number depending on cell types and tissues (Campbell et al., 2012). None of the sequencing studies cited above have considered the percentage of methylated sites respect to total mtDNA molecules present in the cells/tissues analyzed (“methylation heteroplasmy"), but only the percentage of 5 mC respect to total cytosines, as is usually done for the nuclear genome.

The scenario becomes even blurrier when considering the the controversial results on DNMTs localization (**Table 1** and **Table 2**), since DNMT1 presence within mitochondria was demonstrated (Shock et al., 2011; Bellizzi et al., 2013), and then denied by Wong et al. (2013), who in addition observed DNMT3A within mitochondria, but not DNMT3B, which, however, has been detected in these organelles by others (Bellizzi et al., 2013). Therefore, it remains truly unclear which DNMTs might be implicated in mtDNA methylation. Overall, further experiments, focused on both global/site-specific methylation and the functional effects of this modification are necessary to unequivocally demonstrate methylation of the mitochondrial genome, its biological function, and the possible links to pathological conditions.

**Table 1:** Mitochondrial localization of DNA (cytosine-5)-methyltransferases – DNMT1, DNMT3A, and DNMT3B, as evaluated by Western blot, in different human cell types and tissues.

DNA methyltransferase	Mitochondrial localization	Organism	Cell type/tissue	Reference
DNMT1	Yes	Human	HCT116	Shock et al. (2011)
DNMT1	Yes	Human	HEK293	Chestnut et al. (2011)
DNMT1	Yes	Human	HeLa	Bellizzi et al. (2013)
DNMT3A	Yes	Human	HEK293	Chestnut et al. (2011)
DNMT3A	Yes	Human	Frontal cortex	Wong et al. (2013)
DNMT3A	No	Human	HEK293	Wong et al. (2013)
DNMT3B	Yes	Human	HeLa	Bellizzi et al. (2013)

**Table 2:** Mitochondrial localization of DNMT1, DNMT3A, and DNMT3B, as evaluated by Western blot, in different mouse cell types and tissues.

DNA methyltransferase	Mitochondrial localization	Organism	Cell type/tissue	Reference
DNMT1	Yes	Mouse	MEF	Shock et al. (2011)
DNMT1	Yes	Mouse	NSC34 Astrocyte Microglia	Chestnut et al. (2011)
DNMT1	Yes	Mouse	3T3-L1	Bellizzi et al. (2013)
DNMT1	No	Mouse	Adult skeletal muscle	Wong et al. (2013)
DNMT3A	No	Mouse	MEF	Shock et al. (2011)
DNMT3A	Yes	Mouse	NSC34 Astrocyte Microglia	Chestnut et al. (2011)
DNMT3A	Yes	Mouse	Adult skeletal muscle, brain, spinal cord, heart, testes, spleen	Wong et al. (2013)
DNMT3B	No	Mouse	MEF	Shock et al. (2011)
DNMT3B	Yes	Mouse	3T3-L1	Bellizzi et al. (2013)
DNMT3B	No	Mouse	Adult skeletal muscle	Wong et al. (2013)

## Defective DNA Methylation and Neurological Diseases

Altered DNA methylation is a common hallmark of cancer, and 200, 590, and 320 somatic mutations in *DNMT1*, *DNMT3A,* and *DNMT3B*, respectively, have been reported in the catalog of somatic mutations in cancers (COSMIC). Up and down-regulation of DNMTs has also been observed in different types of cancer (Forbes et al., 2008; Subramaniam et al., 2014; Wu et al., 2014).

In addition to cancer, alterations of the DNA methylation machinery also cause a few neurodegenerative and neurodevelopmental diseases, some of these recently described thanks to whole-exome sequencing (Klein et al., 2011; Winkelmann et al., 2012; Tatton-Brown et al., 2014). Genetic defects may produce an epigenetic deregulation at different levels, affecting both the enzymes responsible for *de novo*/maintenance methylation (DNMT3/DNMT1), or proteins with a role in the recognition and binding of CpG methylated sites (MBDs, MeCP2; Weissman et al., 2014). As mentioned previously, in Klein et al. (2011) and Winkelmann et al. (2012), two independent exome-sequencing studies revealed mutations in the RFTS domain of *DNMT1* gene causing two neurodegenerative disorders with overlapping features: hereditary sensory HSN1E and ADCA-DN. More recently, *de novo* mutations in *DNMT3A* gene affecting functional conserved domains of the protein, have been identified by exome-sequencing in patients with overgrowth disorders (Tatton-Brown et al., 2014), resembling clinical features of histone defects and imprinting disorders (Weissman et al., 2014).

Besides primary epigenetic defects, deregulation of DNA methylation may also influence the pathogenesis of other neurodegenerative disorders, like amyotrophic lateral sclerosis (ALS) or Alzheimer disease (AD), Parkinson disease (PD), or neurodevelopmental diseases, such as Down syndrome (DS; Lu et al., 2013). Global DNA hypomethylation in AD has been evidenced in a post-mortem study of monozygotic twins discordant for AD (Mastroeni et al., 2009) and later confirmed by others (Sung et al., 2011; Chouliaras et al., 2013), whereas global hypermethylation has been described in other cases (Bakulski et al., 2012; Rao et al., 2012; Coppieters et al., 2014). In contradiction, Lashley et al. (2014) excluded alterations in global DNA methylation and hydroxymethylation in AD. Hypomethylation of specific genes related to AD (i.e., PSEN1, NEP, BIN1) have also been observed (Lu et al., 2013; Yu et al., 2014). An additional link between DNA methylation and AD came from the association of the chromosome location of *DNMT1* (19p13.2) with familial late-onset AD (FLOAD; Wijsman et al., 2004), although sequencing of exon 20 and 21 of *DNMT1* in 364 FLOAD cases failed to identify pathogenic mutations in these regions, but only known polymorphisms with expected minor allele frequencies based on European HapMap data (Klein et al., 2013).

Hypomethylation of intron 1 of the α-synuclein (*SNCA*) gene might be relevant for PD pathogenesis, inducing an increased expression of α-synuclein in the substantia nigra of PD patients and possibly contributing to the Lewy Body formation (Jowaed et al., 2010; Matsumoto et al., 2010; Lu et al., 2013). Moreover, low levels of nuclear DNMT1 in post-mortem brain of PD patients have been observed, indicating a possible link between *SNCA* hypomethylation and the methylase (Desplats et al., 2011).

DNA methylation seems to have a role also in the pathogenesis of ALS, as proposed by Chestnut et al. (2011), by demonstrating that the levels of DNMT3A, DNMT1, and 5 mC are increased in motor neurons of ALS affected patients. Furthermore, abnormal mtDNA methylation has been found in ALS (Wong et al., 2013) and also in patients affected by Down’s syndrome (Infantino et al., 2011), and for both diseases a mitochondrial dysfunction has been documented (Valenti et al., 2011; Cozzolino et al., 2013).

Based on the mounting evidence of mitochondrial dysfunction in AD and PD, including organelle bioenergetics, dynamics, and quality control (Schon and Przedborski, 2011; Burté et al., 2015), DNA methylation may influence the pathogenesis of these neurodegenerative diseases, acting both on nuclear and mitochondrial DNAs. Moreover, mitochondria participate in the production of the universal methyl donor SAM, through synthesis of ATP and folate, whose deficiency, together with high levels of homocysteine, have been associated with dementia, and neurodegenerative diseases, including AD and PD (Iacobazzi et al., 2013; Ansari et al., 2014). To the best of our knowledge, mtDNA methylation has never been investigated in AD and PD, and neither methylation of nuclear genes encoding for mitochondrial proteins.

## ADCA-DN and HSN1E: Defective Methylation Diseases with Mitochondrial Involvement

Hereditary sensory neuropathy with dementia and hearing loss (OMIM 614116), an adult-onset neurodegenerative disorder, is the first Mendelian inherited “methylopathy" identified due to mutations in the *DNMT1* gene affecting the RFTS domain (Klein et al., 2011). Shortly after, another adult-onset neurodegenerative disease, ADCA-DN (OMIM 604121), has been associated to mutations in *DNMT1* located in the same functional domain (Winkelmann et al., 2012). HSN1E and ADCA-DN have been initially considered as two distinct clinical entities, but more recently the evidence of overlapping clinical features, often subclinical, has emerged, strongly suggesting that they can be better considered as phenotypes belonging to the same neurodegenerative spectrum (Moghadam et al., 2014).

Hereditary sensory neuropathy with dementia and hearing loss is a severe disorder characterized by both central and peripheral nervous system involvement, with peripheral neuropathy leading to extremity injuries and infections, frequently needing amputations, severe early onset hearing loss, and middle-age dementia (Hojo et al., 1999; Klein et al., 2011). By a whole-exome sequencing analysis, two heterozygous mutations in *DNMT1* have been identified as the genetic cause of HSN1E in four unrelated families from different geographical area (Klein et al., 2011). A point mutation leading to the p.Tyr511Cys (NP_001124295.1) aminoacid substitution was found in three families, whereas three nucleotide changes causing the substitution of two contiguous aminoacids, p.Asp506Glu-Pro507Arg (NP_001124295.1) were found in the fourth pedigree of this study; both mutations were located in exon 20 and affected the RFTS domain of DNMT1 (Klein et al., 2011). Through an accurate functional investigation, Klein et al. (2011) demonstrated that these mutations cause a premature degradation of the protein, reduced methyltransferase activity, and impaired binding to heterochromatin in G2 phase, leading to global DNA hypomethylation and site-specific hypermethylation.

The abnormal status of DNA methylationinduced by the p.Tyr511Cys mutation has been thoroughly analyzed by whole-genome bisulfite sequencing in three pairs of HSN1E patients compared to gender and age-matched siblings, providing an evaluation of methylation at single base-resolution (Sun et al., 2014). The results showed prevalent hypomethylation in intergenic regions and around the transcription start sites, with the highest reduction of methylation in chromosomes X and 18 (Sun et al., 2014). Furthermore, using the Ingenuity Pathway Analysis, the differentially methylated regions identified were linked to “neurological disease," including progressive neuropathy, PD, AD, ALS, narcolepsy (NC), “psychological disorders," “skeletal and muscular disorders" and “cancer," whereas the most compromised pathway resulted the NAD^+^/NADH metabolism, which is also implicated in neurodegeneration (Sun et al., 2014).

In 2013 two additional cases of HSN1E with psychiatric manifestations and seizures were identified, again caused by mutations in the exon 20 of *DNMT1*, one of them previously identified (p.Tyr511Cys), and one previously unreported mutation p.Tyr511His (NP_001124295.1), affecting both the same amino acid residue in the RFTS domain (Klein et al., 2013). Two other HSN1E families has been described in 2014, presenting mutations in exon 20 of *DNMT1*: one point mutation (p.Pro506Arg, NP_001124295.1) hitting the same amino acid position of another previously reported case (Klein et al., 2011), and a novel trinucleotide deletion (p.Lys521del, NP_001124295.1; Moghadam et al., 2014). The extensive characterization of the HSN1E patients in this latter study showed that subclinical symptoms usually characterizing ADCA-DN, such as NC (without cataplexy and with normal hypocretin-1 level in cerebrospinal fluid) and optic atrophy, may be present in patients affected by HSN1E, highlighting aphenotypic overlap between these two diseases (Moghadam et al., 2014).

Moreover, the strict association between exon 20 and HSN1E has been broken by Yuan et al. (2013) a unique case of HSN1E caused by a novel mutation (p.His569Arg, NP_001124295.1) in exon 21, that is usually associated with ADCA-DN), but still affecting the RFTS domain and further remarking the idea of a continuum.

Autosomal dominant cerebellar ataxia-deafness and narcolepsy was first described by Melberg et al. (1995), but the genetic cause has been identified only recently in Winkelmann et al. (2012). ADCA-DN is initially characterized by late-onset NC with or without cataplexy, complicated in later stages of the disease by sensorineural deafness, cerebellar ataxia, and dementia appear (Melberg et al., 1995). Mild polyneuropathy, optic atrophy, epilepsy, psychosis, diabetes mellitus, cardiomyopathy, and progressive cerebral, cerebellar, and brainstem atrophy may also be present (Melberg et al., 1999; Winkelmann et al., 2012; Moghadam et al., 2014). *DNMT1* mutations in exon 21 have been found to cause ADCA-DN (Winkelmann et al., 2012), thus qualifying *DNMT1* as the third mutant leading to genetically determined NC (Peyron et al., 2000; Hor et al., 2011). A previous link between *DNMT1* and NC emerged from a genome-wide study associating NC with the SNP rs4804122, located in a region of high linkage disequilibrium spanning several genes including *DNMT1*, and a weak correlation between this NC-associated allele and lower DNMT1 mRNA expression was also documented in peripheral blood mononuclear cells (Kornum et al., 2011).

Initially, three missense mutations have been identified in four different families, all affecting the RFTS domain: p.Ala570Val, p.Val606Phe, p.Gly605Ala (NP_001124295.1). The authors speculated that these mutations might affect the interaction with other proteins, i.e., HDAC2, or the DNA- binding, being close to three phenylalanines critical for the anchoring of the RFTS domain to the DNA-binding pocket (Takeshita et al., 2011; Winkelmann et al., 2012). Concerning the role of mutant DNMT1 in the pathogenesis of NC in ADCA-DN, hypocretin cells, whose loss cause NC, may be particularly susceptible to altered methylation induced by the *DNMT1* mutations (Winkelmann et al., 2012). Alternatively, the mutations may impair the regulation and differentiation of immune cells possibly promoting an autoimmune reaction (Winkelmann et al., 2012), thus supporting autoimmunity as the main pathogenic mechanism of NC (Fontana et al., 2010). This hypothesis is reinforced by the critical role played by DNMT1for T-cells development, function and survival (Lee et al., 2001; Wang et al., 2013). However, the HLA-DQB1^∗^06:02, which represents the major genetic risk factor for NC (Tafti et al., 2014), was negative in all except for two of the ADCA-DN patients with *DNMT1* mutations (Winkelmann et al., 2012; Pedroso et al., 2013; Moghadam et al., 2014), weakening the autoimmune hypothesis for this disease. An additional case of ADCA-DN due to a novel missense mutation in exon 21 of *DNMT1*, p.Cys596Arg (NP_001124295.1), has been reported; this patient displayed the typical, previously described features of the disease (Pedroso et al., 2013).

Interestingly, HSN1E and ADCA-DN present common features resembling mitochondrial encephalomyopathies, such as sensorineural deafness, optic atrophy, cerebellar involvement, and peripheral neuropathy (Moghadam et al., 2014). Furthermore, a mitochondrial dysfunction at the biochemical level was already documented in skeletal muscle of an ADCA-DN patient in Melberg et al. (1995). In addition, the alteration of NAD^+^/NADH-related pathways that emerged from the methylome study on HSN1E (Sun et al., 2014) also reinforces the possibility of mitochondrial dysfunction in the complex pathogenesis of this disorder.

Furthermore, hypomethylation of nuclear genes with mitochondrial function has been observed in HSN1E patients compared to sex and age-matched controls, although a statistical significance was not reached (Sun et al., 2014).

In conclusion, 10 different mutations in exons 20 and 21 of the *DNMT1* gene have been identified to date, six causing HSN1E and four causing ADCA-DN, two distinct diseases now considered as clinical phenotypes of the same disease spectrum (Moghadam et al., 2014). In fact, all the mutations affect the RFTS domain, which has a regulatory function for the DNMT1 activity. In particular, mounting evidence demonstrate that this domain has an auto-inhibitory role (Bashtrykov et al., 2014) and it has been suggested that its deletion activates DNMT1 for euchromatic DNA-binding, but at the same time decreases heterochromatin binding probably through a missing protein interaction, thus producing passive DNA demethylation (Wu et al., 2014). Moreover, the RFTS deletion seems to produce a methylation status typical of cancer, with global hypomethylation and promoter hypermethylation, and additional 26 mutations in this domain have been found in different tumors (Forbes et al., 2008; Wu et al., 2014).

Open questions still have to be addressed. For example, it is unclear whether mutations in exon 20 and 21 of *DNMT1* act through the same pathogenic mechanism, and the methylation status of ADCA-DN patients has not been investigated yet. Furthermore, although the altered methylation pattern observed in HSN1E patients resembles that of tumorigenesis (Sun et al., 2014) and loss of function of RFTS domain enhances tumorigenicity (Wu et al., 2014), none of the *DNMT1* mutant patients seem to develop cancer. Lastly, the possible role of mitochondrial dysfunction deserves to be unraveled by functional studies, to shed light on the potential role of mito-epigenetics (from both nuclear and mitochondrial genomes) in the pathogenic mechanisms of *DNMT1* mutations. Tackling all these open questions will be crucial to understand the pathogenesis of *DNMT1* related neurodegeneration, including the narcoleptic features, but ultimately will also help to resolve the question of mtDNA methylation.

## Final Remarks

The fascinating field of epigenetic regulation of gene expression is fast evolving, as well as the implications in human pathology. One point highlighted by reviewing the state of art of this field, and prompted by the recent identification of neurodegenerative disorders due to pathogenic mutations in the *DNMT1* gene, is the role played by the mitochondrial methylome as a whole, includes obviously the tissue and cell-specific methylation pattern of the nuclear mitochondrial proteome (about 1500–2000 nuclear genes), and the still very controversial existence of mtDNA methylation. This latter longstanding question needs to be resolved, and the possible biological function played by mtDNA methylation in cell physiology may add to the complex inter-genomic dialog between nuclear and mitochondrial DNAs. The rapidly evolving NGS approaches should allow the resolution of the controversies, in conjunction with functional studies, paying attention to the peculiar features of the mitochondrial genome, which include gene organization, transcription, and replication, and most importantly its multicopy nature. The nuclear counterpart represented by the growing list of nuclear genes implicated in mitochondrial biology is equally crucial for understanding the complexities of cancer and neurodegeneration, now fuelled by the existence of an ideal model to investigate, the DNMT1-related human diseases.
